# IL‐7 germline variant: setting the stage for immune‐related adverse events

**DOI:** 10.1002/1878-0261.13392

**Published:** 2023-02-24

**Authors:** Hussein Issaoui, Jean‐Ehrland Ricci

**Affiliations:** ^1^ Université Côte d'Azur, INSERM, C3M Nice France; ^2^ Equipe Labellisée Ligue Contre le Cancer Nice France

**Keywords:** germline variant, immune checkpoint inhibitors, immune‐related adverse events, interleukin 7, lymphocytes

## Abstract

Treatment with immune checkpoint inhibitors (ICIs) is frequently associated with immune‐related adverse events (irAEs). A new study identified an interleukin 7 (IL‐7) allelic variant—rs16906115—as a major risk factor for the development of ICI‐associated irAEs. This association is of great significance as it indicates that germline genetic variants influence the occurrence of irAEs, thus opening a new avenue for identifying high‐risk patients to enable better management of ICI therapy and associated irAEs.

## Graphical abstract

During immune checkpoint inhibitor (ICI) therapies, some patients experience inflammatory side effects called immune‐related adverse events (irAEs). The authors of the new research discovered an IL‐7 allelic variant, whose presence in these patients' lymphocytes increases lymphocyte stability and leads to an active host immunity, as a major risk factor of developing ICI‐associated irAEs.

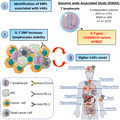

**Abbreviations**
APC: antigen‐presenting cells
CTLA‐4: cytotoxic T‐lymphocyte‐associated protein 4
GWAS: genome‐wide association study
ICI: immune checkpoint inhibitors
IL‐7: interleukin 7
irAEs: immune‐related adverse events
PD‐1: programmed cell death protein 1
PD‐L1: programmed death‐ligand 1
SNP: single‐nucleotide polymorphism

In contrast to immunocytokine therapies, ICIs have made a breakthrough in cancer therapy over the past decade [1]. ICIs work by unblocking the inhibitory signaling effect of immunomodulatory receptors expressed by immune or cancer cells (such as CTLA‐4 and PD‐1 or PD‐L1), which usually allow cancer cells to escape destruction. This inhibition will lead to a strong activation of the immune system orchestra, particularly T cells, and consequently to a more effective antitumor response [2, 3, 4, 5].

Despite the significant success of ICIs in improving survival in some patients, they are frequently associated with adverse events (from rash to severe colitis and myocarditis) and toxicities that can be fatal in some cases. Thus, overactivation of the immune system may act as a ‘poisoned chalice’ as it may also lead to the development of inflammatory side effects, called irAEs, which may lead to discontinuation of ICI therapy [6, 7, 8]. Over the years, considerable effort has been made to stratify the patients that will respond well to ICI therapy and those that will be less susceptible to irAEs. However, the underlying relationship between the emergence of irAEs and ICI therapy remains unclear. One of the proposed hypotheses is that the composition of patients' microbiota is associated with the development of irAEs and the efficacy of ICIs [9, 10]. Also, the influence of host germline genetic factors has been associated with drug exposure and patient susceptibility to toxicity of different chemotherapeutic agents [11]. Previous studies have shown a correlation between polygenic germline risk of autoimmune conditions and the emergence of thyroid and skin irAEs linked to ICIs [12]. However, to date, no individual genetic variants have been linked to irAEs. In the December 2022 issue of *Nature Medicine*, Gusev and colleagues identified a germline variant of IL‐7 as a crucial regulator of lymphocyte stability and a critical predictor of irAEs occurrence [13].

First, by performing a genome‐wide association study (GWAS) on 1751 patients with 12 cancer types treated with ICI, Gusev and colleagues classified each patient according to irAEs outcomes after initiation of ICI treatment (either ‘high‐grade’ or ‘all‐grade’ irAEs). After verifying that the power analysis of their samples was sufficient to detect large effect variants, they identified three genome‐wide significant loci linked to all‐grade irAEs (but none with ‘high‐grade’ irAEs): one association at chr4p15, one near the interleukin 22 receptor subunit alpha 1 (IL22RA1), and the third one near the IL‐7 at Chr8q21 [13]. None of these variants were associated with overall survival nor with death without irAEs.

The authors investigated the effect of factors that could modify the link observed between IL‐7 variant and irAEs occurrence and demonstrated that the observed effects were specific to ICIs treatment, as none of them were strongly related to age, sex, or even previous autoimmune diseases, underscoring the involvement of these germline variants in irAEs [13]. The link observed between the rs16906115 variant near IL‐7 and irAEs occurrence was significantly reproduced in two additional independent cohorts, as opposed to the two other SNP identified [13]. Although the noted effect of IL‐7 SNP was not linked to the OS of patients under ICIs, this SNP also uncovered an antitumor response beyond the ICI therapy. The authors strikingly discovered a persistent significant association between the IL‐7 variant and OS of patients treated with conventional therapies [13].

To uncover the link between the IL‐7 locus and irAEs, the authors integrated quantitative trait loci (QTLs) and RNA sequencing analysis to their GWAS data. Remarkably, they identified a novel 70‐base pair ‘cryptic exon’ that was highly activated in patients carrying the IL‐7 SNP compared with noncarriers. Cryptic exons are splicing variants introducing modifications of the resulting mRNA, such as frameshift codons. Using a *de novo* isoform reconstruction method, they were able to reveal a new IL‐7 transcript that was highly expressed in lymphoblastoid cell lines, consistent with the function of IL‐7 in lymphocyte development [14]. This increase in expression was also associated with an increased co‐expression of IL‐7 and IL‐7 receptor, suggesting that the new IL‐7 transcript may act as an IL‐7 stabilizer or an IL‐7R binding enhancer [13]. Based on previous knowledge about the role of IL‐7 in lymphocyte homeostasis [14], the authors finally investigated the effect of the IL‐7 variant on peripheral blood lymphocyte counts at the initiation of ICIs and the onset of irAEs in two different cohorts. Indeed, they were able to show a significant decrease in the lymphocyte count in patients not carrying the IL‐7 variant compared with carriers of this variant [13]. Thus, these results suggest a consistent function of the IL‐7 variant on lymphocyte homeostasis and irAEs incidence during ICIs therapy.

In GWAS association studies, the power of the study is often impaired by the sample size analyzed and a lack of independent validation. In this study, by Gusev and colleagues [13] this IL‐7 variant was associated with irAEs using a large sample size (> 1000 patients) and two independent validation cohorts making it as the first genetic variation robustly associated with irAEs. In the future, an even larger, well‐powered study should uncover new germline genetic variants influencing different grades of irAEs. Indeed, it will be important to identify markers for ‘high‐grade’ irAEs, as lower‐grade irAEs have been associated with better outcome, indicating that some toxicity may be acceptable and may be indicative of an efficient anticancer immune response. The type of ICI treatment (e.g., single anti‐PD‐1/PD‐L1 or anti‐CTLA‐4 therapy or combination regimens) will have a large influence on irAEs. In this study, the vast majority of patients received single‐agent PD‐1/PD‐L1 treatment. As such, it remains to be determined whether the IL‐7 variant is associated with CTLA‐4‐related irAEs or a combination of ICI regimen‐driven irAEs, which are known to be associated with higher incidence of ‘high‐grade’ irAEs. Finally, it will also be important to determine whether pre‐existing autoantibodies or elevated serum cytokines may represent valid biomarkers. This could provide mechanistic insights into patient susceptibility to irAEs since an effective predictive model of irAEs may allow for more personalized treatment enabling better management or prediction of ICI treatment‐induced toxicities.

## Conflict of interest

The authors declare no conflict of interest.
